# Covid-19 and Cancer Plastic Surgery

**DOI:** 10.29252/wjps.9.3.354

**Published:** 2020-09

**Authors:** Muhammad Ahmad

**Affiliations:** Department of Plastic and Hair Restorative Surgery, Aesthetic Plastic Surgery and Hair Transplant Institute, Islamabad, Pakistan

**Keywords:** COVID-19, Cancer, Plastic Surgery

**DEAR EDITOR**

The pandemic of COVID-19 has taken the world by storm. Practically no part of the world is free of the disease. It has affected more than 2 million people with over 146,000 worldwide mortalities.^1^ The disease has no treatment, no cure and no vaccination. The overwhelming number of sick patients has driven the health care systems to the limit and any surge in the patients could result in collapsing of the health care system. Majority of the patients have resulted in bed occupancy for more than weeks. Moreover, the ailments in health-care providers are another major issue. By flattening the curve, the number of mortalities is reduced and the system manages to cater for the sick patients. However, flattening the curve results in prolonging the duration of the disease; as well. 

According to WHO facts sheet, there were 9.6 million deaths from the cancer alone worldwide,^2^ and there were 17 million new cancer cases in 2018. Many of these patients require surgical excisions.^3^ In the time of COVID-19 pandemic, the health care workers and places are pre-occupied, leaving little to no space for these patients. The plastic surgeons managing these patients should be careful as there is no mass screening test available to distinguish between a possible carrier and asymptomatic COVID-19 patient. Therefore, for all elective surgeries, a new protocol should be ensured. This protocol must include the Personal Protective Equipment (PPE) gowns, surgical masks, goggles and face-shields (Figure 1) and the surgeons should assume every visiting patient as a potential carrier. By maintaining the standard protocols, they can treat these patients and protect themselves and the staff as well.

**Fig. 1:**
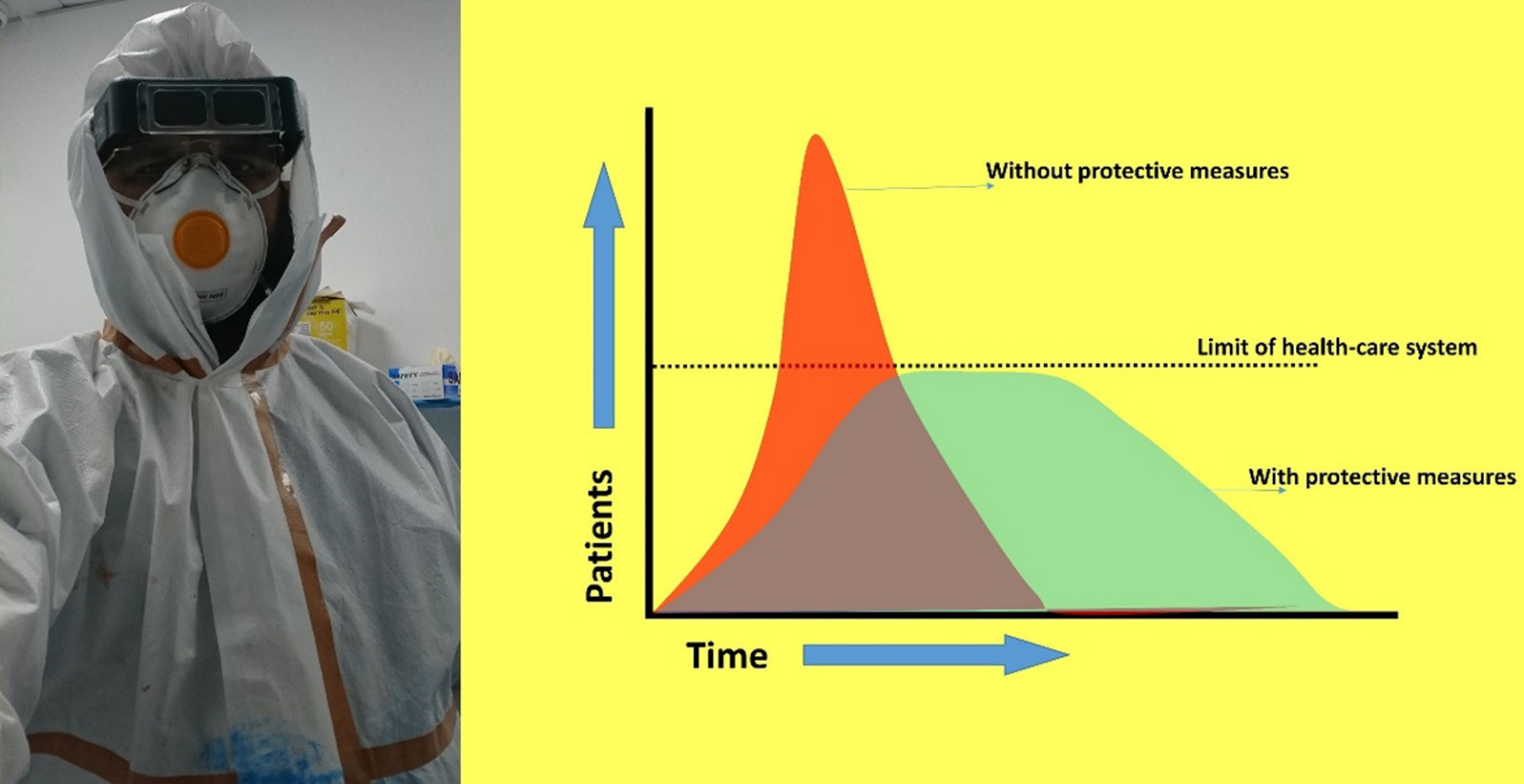
Protective measures and flattening of the curve

## CONFLICT OF INTEREST

The authors declare no conflict of interest.
